# Genetic Analyses of Flower Main Traits from Two Pitayas and Their Progenies: A Cactus Plant

**DOI:** 10.3390/plants13050699

**Published:** 2024-02-29

**Authors:** Tiantian Zhang, Kangmin Xing, Jiayi Chen, Irfan Ali Sabir, Kamran Shah, Jiaxuan Chen, Zhike Zhang, Jietang Zhao, Guibing Hu, Yonghua Qin

**Affiliations:** 1 Guangdong Provincial Key Laboratory of Postharvest Science of Fruits and Vegetables, College of Horticulture, South China Agricultural University, Guangzhou 510642, China; 17863608557@163.com (T.Z.); X15975593123@163.com (K.X.); chenjiayi98@stu.scau.edu.cn (J.C.); irfanalisabir@sjtu.edu.cn (I.A.S.); kamranshah801@scau.edu.cn (K.S.); jxchen0127@163.com (J.C.); poloky2@163.com (Z.Z.); zhaojietang@gmail.com (J.Z.); guibing@scau.edu.cn (G.H.); 2 Key Laboratory of Biology and Genetic Improvement of Horticultural Crops (South China), Ministry of Agriculture and Rural Affairs, College of Horticulture, South China Agricultural University, Guangzhou 510642, China

**Keywords:** breeding, flower traits, genetic variation, mixed genetic model, pitaya, reciprocal crossing

## Abstract

Elucidation of the genetic foundation governing crucial traits in pitaya flowers is imperative for enhancing both the ornamental and economic values. In this study, the dynamic variation in flower genetics, segregation variation patterns, and a mixed inheritance model of the major and multigene flower traits of ‘Dahong’ and ‘Honghuaqinglong’ pitayas and their progenies were explored. The results showed that the main traits of flowers exhibited varying degrees of variation among the reciprocal F1 hybrids, with the data exhibiting the characteristics of quantitative traits. The betalain content, petal number, and stigma number exhibited values below the median values of the parents, suggesting a genetic inclination towards lower values. Perianth width, calyx tube width, petal number, and stigma number had the same genetic effects and significant correlation. Stigma-related traits had a clear maternal inheritance tendency. The heritability of flower length, stigma relative to anther distance, and petal betalain content was governed by two pairs of additive-dominant major genes. Perianth width, calyx tube width, petal number, and stigma number all conformed to the model of two pairs of equal-additive-dominant major genes. This study provides valuable information for parental selection, cross-breeding, and the enhancement of pitaya varieties to meet market preferences and environmental conditions.

## 1. Introduction

The pitaya belongs to the *Hylocereus* genus of the Cactaceae family and originated in the tropical regions of Mexico and Central America [1]. The pitaya has gained significant attention in recent years due to its ornamental and nutritional value, presenting substantial potential in both domestic and international markets [2]. Pitaya fruit contains betalain, dietary fiber, protein, vitamins, and minerals [3]. Pitaya fruit peel can be processed to food packaging and coatings [4], the seeds can be used to extract antioxidant-rich oil [5,6]. According to the peel and pulp color, pitayas can be divided into four categories: *H. polyrhizus*/*H. monacanthus*/*H. costaricensis* (red peel with scales and red pulp), *H. undatus* (red or yellow peel with scales and white pulp), *H. megalanthus* (yellow peel without scales and with white pulp), and *H. stenopterus* (green peel with scales and white pulp) [3,7]. Except for *H. megalanthus*, the other three *Hylocereus* species are diploid with a chromosome number of 2n = 22. *H. megalanthus* is a tetraploid species (2n = 44) [8], which suggests its formation through natural hybridization between closely related diploid taxa. This polyploidy impacts fruit size, seed number, and pollen viability due to chromosomal disjunction anomalies [9]. In recent years, great progress has been made in pitaya new cultivar breeding, with good fruit quality through seedling selection, bud mutation, and cross-breeding [10,11,12,13,14]. Cross-breeding, particularly as an effective approach for enhancing fruit varieties, has played a crucial role in the cultivation and dissemination of new pitaya varieties [15].

Flowers play crucial roles in insect pollination, fruit setting, and yield [16]. Multiple flower colors with a big size will contribute to attracting insects to pollinate. In addition to flower color and size, the relative position of stigmas and anthers can also influence pollination [17]. Currently, pitaya cultivars in large-scale commercial cultivation have white flowers. Many cultivars from *Hylocereus* are self-incompatible and the stigmas are higher than the anthers [9]. Metaxenia may influence fruit quality in cross-pollinated vine cacti, thus, artificial cross-pollination is essential to increase fruit setting and fruit weight [18]. Pitaya flowers, also called Moonflowers, Queen of the Night, or the Lady of the Night, only open at night and close at daybreak, with each flower lasting only one night [19,20]. Hand pollination in pitaya cultivation is characterized by inefficiency, labor intensiveness, and time-consuming procedures. Consequently, the development of a new pitaya flower breed holds significant importance to address these challenges.

Previous studies on pitaya were mainly focused on nutritional composition [21,22], biological activities [23,24], cultivation [25], diseases [26,27], and betalain biosynthesis [28]. Recently, 13 differentially expressed genes (DEGs) involved in light-induced flowering and different flower growth stages of the pitaya were identified at the transcriptomic level [29,30]. Currently, there are some studies on genetic patterns of pitaya fruit, however, the genetic law of flower traits remains to be elucidated. In the present study, characterizations of the flower morphology and segregation patterns of F1 progenies from ‘Dahong’ × ‘Honghuaqinglong’, ‘Honghuaqinglong’ × ‘Dahong’, and their parents were analyzed. The aim of this study is to explore the genetic law of pitaya flower traits and provide a foundation for parent selection in pitaya breeding. 

## 2. Results

### 2.1. Hybrid Authenticity Verification

**Figure 1:**
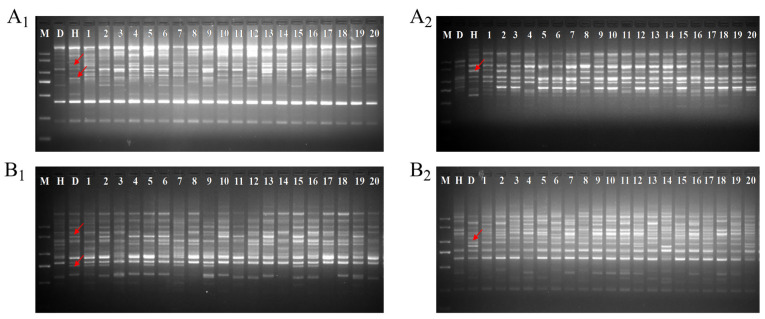
Hybrid identification of part F1 progenies using primers of SCoT-42 (**A_1_**), SCoT-19 (**A_2_**), SCoT-58 (**B_1_**), and SCoT-63 (**B_2_**). M, 2000 bp marker; D, ‘DH’ pitaya; H, ‘HHQL’ pitaya; 1–20, F1 progenies. Arrows indicated specific bands of the male parent.

The SCoT molecular marker was used to verify authentic hybridization of 20 F1 progenies randomly selected from the ‘DH’ × ‘HHQL’ and ‘HHQL’ × ‘DH’ cross combinations. Four SCoT markers showed polymorphism between parental lines. In the ‘DH’ × ‘HHQL’ cross combination, the SCoT primers SCoT-42 (Figure 1A_1_) and SCoT-19 (Figure 1A_2_) produced specific bands in the male parent (‘HHQL’), and these specific bands were present in the F1 progenies. Similarly, in the ‘HHQL’ × ‘DH’ cross combination, the SCoT primers SCoT-58 (Figure 1B_1_) and SCoT-63 (Figure 1B_2_) showed a polymorphic band in the male parent and F1 progenies. These SCoT markers were successfully used to confirm the hybridity of the ‘DH’ × ‘HHQL’ and ‘HHQL’ × ‘DH’ cross combinations.

### 2.2. Petal Color and Stigma Fork Traits 

**Figure 2:**
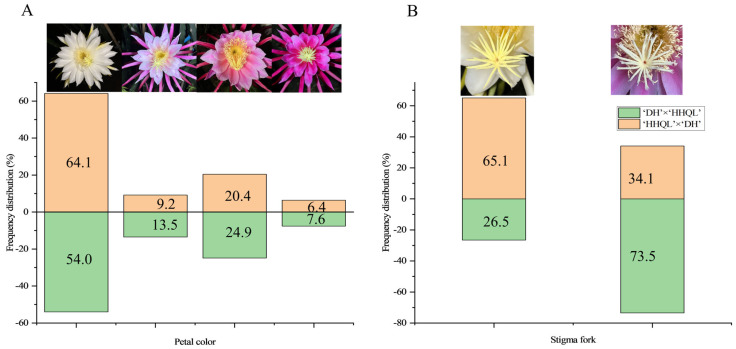
Flower traits of F1 progenies from ‘DH’ × ‘HHQL’ and ‘HHQL’ × ‘DH’ cross combinations. (**A**) Petal color; (**B**) Stigma fork.

Abundant genetic variations were detected in the petal color and stigma fork of F1 progenies from ‘DH’ × ‘HHQL’ and ‘HHQL’ × ‘DH’ cross combinations (Figure 2). The petal colors of ‘DH’ × ‘HHQL’ and ‘HHQL’ × ‘DH’ F1 progenies had different separation ratios and the largest proportion of petal color was white. There were 64.1%, 9.2%, 20.3%, and 6.4% white, pale pink, pink, and red petals, respectively, in F1 progenies of the ‘DH’ × ‘HHQL’ compared to 54.0%, 13.5%, 24.9%, and 7.6% for ‘HHQL’ × ‘DH’, respectively. In the ‘DH’ × ‘HHQL’ cross combination, 26.5% of F1 progenies had a stigma fork compared to 73.5% F1 progenies with a stigma fork for the ‘HHQL’ × ‘DH’ cross combination. 

### 2.3. Distributions of Main Flower Traits 

**Figure 3:**
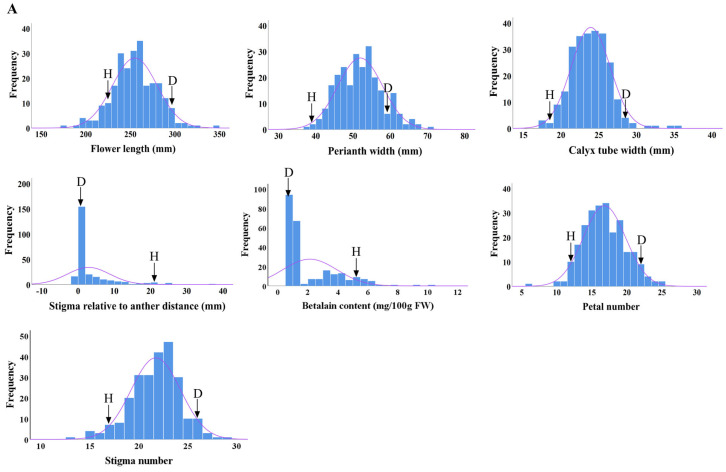

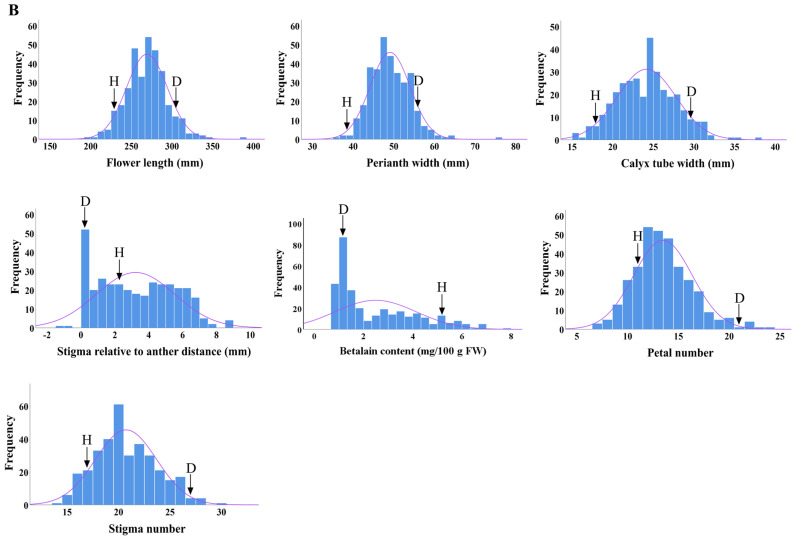
Frequency distribution of ‘DH’ × ‘HHQL’ (**A**) and ‘HHQL’ × ‘DH’ (**B**) cross combinations, ‘D’ indicates ‘DH’, ‘H’ indicates ‘HHQL’.

The distributions of seven main flower traits in the F1 progenies from ‘DH’ × ‘HHQL’ and ‘HHQL’ × ‘DH’ cross combinations were analyzed. The phenotypic values for flower length, perianth width, calyx tube width, number of petals and stigmas, betalain content of the petals, and stigma–anther relative position in most F1 progenies were found to be within the range observed for the ‘DH’ and ‘HHQL’ pitayas, with a few individuals displaying transgressive traits. As shown in Figure 3, flower length, perianth width, calyx tube width, number of petals and stigmas showed a normal distribution compared to a skewed distribution for the betalain content of petals and stigma–anther relative position. Those results suggested that flower length, perianth width, calyx tube width, number of petals and stigmas, betalain content of petals, and stigma–anther relative position are quantitative traits. 

### 2.4. Correlation Analyses of Flower Traits

**Figure 4:**
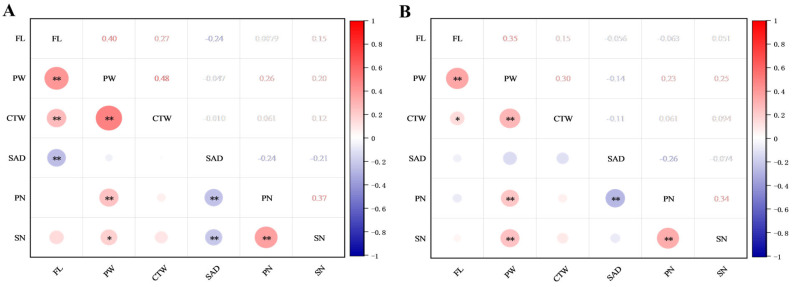
Correlation analyses of flower traits in F1 progenies of ‘DH’ × ‘HHQL’ (**A**) and ‘HHQL’ × ‘DH’ (**B**) cross combinations. FL, flower length; PW, perianth width; CTW, calyx tube width; SAD, stigma relative to anther distance; PN, petal number; SN, stigma number. * *p* < 0.01, ** *p* < 0.001.

Correlations of the seven flower traits in the F1 progenies of ‘DH’ × ‘HHQL’ (A) and ‘HHQL’ × ‘DH’ (B) cross combinations were analyzed (Figure 4). A significant positive correlation was observed between flower length, perianth width, calyx tube width, perianth width, petal number, and stigma number. Stigma relative to anther distance had a moderately negative correlation with the other traits. Those results suggested that larger flowers have more petals and a higher stigma number and the stigma relative to anther distance might have evolved independently.

### 2.5. Genetic Analyses of Flower Traits

As shown in Table 1, flower length, perianth width, calyx tube width, distance between stigma and anther, petal betalain content, petal and stigma number had different degrees of variation. Flower length, perianth width, calyx tube width, petal and stigma number exhibited similar patterns of variation. They all had lower coefficients of variation and a higher genetic transmission ability, indicating that these traits are less influenced by environmental factors and can be stably inherited. Significant variability in stigma–anther distance and petal betalain content were detected among the F1 progenies of ‘DH’ × ‘HHQL’ and ‘HHQL’ × ‘DH’ cross combinations. 

The mid-parent heterosis (Hm) of the calyx tube width, petal betalain content, petal and stigma number showed significant levels ranging from −0.28 to −9.68 in ‘DH’ × ‘HHQL’, and from −0.94 to −9.85 in ‘HHQL’ × ‘DH’ cross combination, indicating there is a significantly negative heterosis for flower size. F1 progenies of the ‘HHQL’ × ‘DH’ cross combination showed a predominance of positive heterosis rates in traits in terms of flower length, perianth width, and stigma–anther distance, with stigma–anther distance displaying the highest rate of 190%, suggesting there is favorable mid-parent heterosis.

### 2.6. Genetic Analyses of Main Genes and Multigene of Flower Traits

#### 2.6.1. Suitability Test of the Optimal Genetic Model for Flower-Related Traits

A genetic analysis was performed using the mean value of seven flower traits (Table 2). According to the AIC minimum criterion and taking the flower length as an example, the 2MG-AD and 2MG-EA models, which have the minimum AIC value or a relatively small AIC value, were selected as alternative optimal models for the subsequent suitability test. U1^2^, U2^2^, U3^2^, _n_W^2^, and D_n_ were used to test the suitability of the alternative models (Table 3). The results showed that the 2MG-AD model with the lowest AIC value was the optimal model for the flower length, which was controlled by two pairs of additive-dominant major genes. The best-fit model for petal color was the 2MG-AD model. The most suitable model, i.e., the 2MG-EA model for petal number and stigma number, was controlled by two pairs of equal additive major genes. The optimal genetic model of perianth width, calyx tube width, and stigma relative to anther distance was different between the two cross combinations. Since the number of F1 progenies from the ‘HHQL’ × ‘DH’ cross combination is larger than that of the ‘DH’ × ‘HHQL’ cross combination, the results of ‘HHQL’ × ‘DH’ cross combination prevail. Therefore, the 2MG-EA model was selected as the optimal model for perianth width and calyx tube width, and the 2MG-AD model for stigma relative to anther distance (Table 3).

#### 2.6.2. Genetic Parameter Estimation of Flower-Related Traits under the Optimal Genetic Model

The genetic parameters of the optimal genetic model are shown in Table 4. For flower length, the heredity was controlled by two pairs of additive-dominant major genes; the heritability of the major genes was 91.11% and 0% in the ‘DH’ × ‘HHQL’ and ‘HHQL’ × ‘DH’ cross combinations, respectively. The first pair of major genes was positive and the second pair of major genes was negative. Similarly, stigma relative to anther distance and petal betalain content were consistent with the model of two pairs of additive-dominant major genes; the heritability of the major genes was 86.45% and 46.40%, respectively. Perianth width, calyx tube width, petal number, and stigma number all conformed to the model of two pairs of equal-additive-dominant major genes. In the ‘DH’ × ‘HHQL’ cross combination, the additive effects of the first pair of genes were 6.674, 2.220, 1.49, and 0.74, respectively, while the heritability of the major genes was 36.08%, 0%, 27.77%, and 100%, respectively. 

## 3. Materials and Methods

### 3.1. Plant Materials

**Figure 5:**
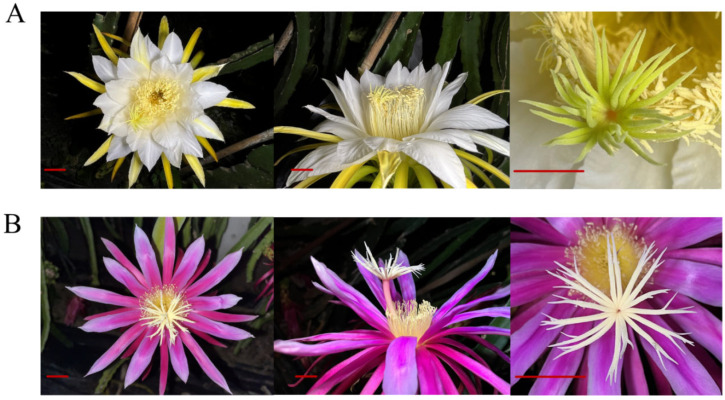
Flowers of the ‘DH’ (**A**) and ‘HHQL’ (**B**) pitayas. Bar = 2 cm.

‘Dahong’ (DH) (*H. monacanthus*), ‘Honghuaqinglong’ (HHQL) (*H. stenopterus*), F1 progenies of 249 ‘DH’ × ‘HHQL’ and 340 ‘HHQL’ × ‘DH’ were used as materials. ‘DH’ × ‘HHQL’ and ‘HHQL’ × ‘DH’ are regarded as positive and negative cross combinations, respectively. All materials were planted in the pitaya germplasm resource at the College of Horticulture, South China Agricultural University (113.36° N, 23.11° E), China. ‘DH’ and ‘HHQL’ were from the same species but with distinct genotypes and phenotypes. ‘DH’ is a compatible cultivar with white petals while ‘HHQL’ is an incompatible pitaya cultivar with red petals (Figure 5). The stigma and petal numbers of the ‘DH’ pitaya are significantly higher than those of the ‘HHQL’ pitaya. Compared with the ‘DH’ pitaya, a stigma split of the ‘HHQL’ pitaya is present. The stigma of the ‘HHQL’ pitaya is significantly higher than that of the anthers while the stigma–anther relative position of the ‘DH’ pitaya is equal (Figure 5 and Table 5).

### 3.2. Acquirement of Hybrids

Stamens from flowers of the ‘DH’ and ‘HHQL’ pitayas were removed at around 3 p.m. before bloom, and then entire flowers were covered with non-woven fabric bags until pollination. On the same day at approximately 10 p.m. (when the flowers were fully open), pollen from the paternal plants were collected using a soft brush and used to pollinate the stigmas of the maternal plant. Each mature fruit without peel was placed in a cheesecloth and gently rubbed under tap water until the flesh was fully removed. The seeds were then air dried at room temperature for 48 h after removing aborted seeds floating on the water surface. Seeds were put in a 50 mL centrifuge tube with 40 mL water and placed in a constant temperature shaker at 28 °C, 180 rpm conditions and cultured for 72 h. The water was changed every 12 h. Seeds were mixed with appropriate nursery soil and evenly sown in the seedling tray. After watering thoroughly, the seedling tray was placed in a well-ventilated and dry location. The seedlings were planted to field when they were approximately 30–50 cm in height. 

### 3.3. Authenticity Identification

DNA was extracted using a CTAB Plant DNA Kit (Aidlab Biotechnologies Co., Ltd., Beijing, China) according to the manufacturer’s instructions. Sixteen SCoT primers (Table 6) were used for true hybrid identification [31]. The SCoT-PCR-based reactions were conducted in a 20 μL reaction mixture containing 3.05 μL of template DNA, 2 μL 10× buffer, 2.5 μL MgCl_2_ (25 mM), 1.52 μL dNTPs (2.5 mM), and 5 U/μL *Taq* DNA polymerase. The PCR amplification was performed at 94 °C for 5 min, 94 °C for 50 s, then 56 °C for 1 min, 72 °C for 2 min, followed by 35 cycles, and the final extensions were performed at 72 °C for 10 min.

### 3.4. Investigation of Flower Characteristics

Various characteristics of the flowers of the two parents and their F1 progenies were recorded according to the Guidelines of *Hylocereus* descriptors [32]. Flower length, perianth width, and calyx tube width were measured using a digital vernier caliper (Shanghai Manette Industries (Group) Co., Ltd., DEGUQMNT, Shanghai, China) in the afternoon on the day when the flower will open at night. Number of petals and stigmas, colors of flower petal and stigma, stigma split, and stigma–anther relative position were recorded on the evening of the flower opening. 

### 3.5. Measurement of Betalains

Petals from parental plants and F1 progenies were collected and ground into powder using a rapid grinder in liquid nitrogen. A total of 0.5 g of sample was homogenized with 5 mL 80% aqueous methanol (*v*/*v*) solution. Samples were sonicated for 10 min in an ultrasonic cleaner (SB25-12DT, Ningbo, China) and then stirred for 20 min in darkness at room temperature. Supernatants were collected at 2200 rpm for 10 min, and the residues were subjected to a similar second extraction. The supernatants were measured through spectrophotometry (Infinite M200, Tecan Co, Zurich, Switzerland). Betacyanin contents were calculated by the following equation:

$$Betacyanin\;contents(mg/100\;g\;fresh\;pulps)=\frac{\left(A_{538}\times DF\times W\times V\times 100\right)}{\left(\varepsilon \times P\times L\right)}$$

A_538_ is the absorbance readings noted from the spectrophotometer for betacyanins, DF is a dilution factor, W is the molecular weight (550 g/mol for betalain), V is the pigment solution volume (mL), ε is the molar extinction coefficient (60,000 L/mol·cm for betalain), and L is the length of the cell (1 cm). P is the fresh weight (g). All samples were performed in triplicate [33].

### 3.6. Inheritance Analyses of Flower-Related Traits

The maximum likelihood value (MLV) of various genetic models was obtained by combining the distribution of the phenotype frequency with 11 genetic models based on the phenotypic data of the F1 population. Then, Akaike’s information criterion (AIC) was calculated from the MLV, and the relative optimal model was performed based on the AIC value [34]. Statistical tests, including a Uniformity test (U_2_^1^, U_2_^2^ and U_2_^3^), Smirnov test (nW^2^), and Kolmogorov test (Dn), were used to check the accuracy according to the candidate models. The least squares method was used to estimate the effect value, variance, heritability, and other genetic parameters of the major genes. Segregation Analysis (SEA v2.0) and R v4.2.0 were used to analyze the mixed major gene plus polygene inheritance https://cran.r-project.org/web/packages/SEA/ (accessed on 20 March 2023).

### 3.7. Statistical Analysis

The data were subjected to SPSS 21.0 to calculate the average, standard deviation, and coefficient of variation. Data were tested for a normal distribution using the Kolmogorov–Smirnov test (*p* < 0.05). Correlation heat maps were performed using Origin 2018.

Mid-parent heterosis (Hm) was calculated by the equation:

Hm = F − MP

Mid-parent heterosis rates (R_Hm_) were calculated by the equation:

R_Hm_ = (F − MP)/MP × 100

Genetic transmitting ability (Ta) was calculated using the equation:

$$Ta=\frac{F}{\mathrm{MP}}$$

where F is the mean value of the F1 generation and MP is the mid-parent value [35]. 

All data were analyzed using Excel 2019 and SPSS 21.0, and the difference was statistically significant based on a single sample mean *t*-test. 

## 4. Discussion

### 4.1. The Genetic Effect of Flower Color

The determination of flower color, a pivotal ornamental trait, is intricately linked to the presence of specific pigments. An in-depth exploration of the genetic principles governing flower color provides a foundational basis for the systematic cultivation of novel varieties characterized by diverse and aesthetically appealing flower colors [36]. In this study, reciprocal crosses were conducted between ‘DH’ (white petals) and ‘HHQL’ (red petals) pitayas and the separation and variation of flower color in the F1 progenies were studied. Four distinct flower colors: white, pale pink, pink, and red were observed, indicating an incomplete dominance inheritance pattern, which was consistent with the results of the flower color traits of *Plumbago auriculata* [37] and strawberries [38]. Previous literature illustrated that the genetic mechanisms underlying flower color variations in plants are complex, involving various factors [39,40].

In ornamental plants, the captivating array of flower colors is orchestrated by the intricate interplay and accumulation of four essential groups of pigments: flavonoids, carotenoids, betalains, and chlorophylls. The compositions and contents of these pigments result in a wide variety of flower colors [41,42]. Betalains have the remarkable ability to impart vibrant red–violet and yellow–orange colors to various plant tissues, including leaves, stems, fruits, flowers, roots, and seeds [43]. 

In our current investigation, we observed a direct correlation between the flower color of the F1 progenies and the pigment content. Specifically, a higher concentration of betalain resulted in a deeper and more intense coloration. It is noteworthy that white flowers were predominantly observed in both cross combinations, with a consistent color distribution among the different groups. Betalain contents in most F1 progenies ranged from 1 to 3 mg/100 g FW, suggesting that there is a good relationship between betalain levels and flower color. Notably higher betalain contents were detected in petals (Figure 4), suggesting more potential for flower color variations in the F1 progenies of the ‘DH’ × ‘HHQL’ and ‘HHQL’ × ‘DH’ cross combinations. Results from genetic analysis showed that two gene pairs influence these flower colors, demonstrating additive-dominant effects; similar findings were also obtained in small-flowered chrysanthemums [44].

### 4.2. The Genetic Effect of Flower Types

Flower type is a significant trait that affects mating system evolution and reproductive success. Promoting the evolution of floral morphology toward larger flowers is more favorable for pollinators [45]. Understanding the genetic control model of flower traits is of great significance for breed improvement in plants [46]. Previous studies found that, in peaches, the phenotype of multi-petal flowers is a single recessive trait [47], while the single-petal flower type is controlled by a recessive allele in carnations [48]. In our study, pitaya flower-type related traits, such as perianth width, calyx tube width, petal and stigma number, all followed the 2MG-EA model, indicating that they are controlled by two pairs of equally additive major genes. However, the flower length was controlled by two pairs of additive-dominant major genes, and there was no significant correlation with other traits, suggesting that the flower length may be independently inherited. Therefore, the flower-related traits were most likely controlled by two pairs of major genes (Table 6). Previous studies have shown that plant phenotypic traits are the result of the close interaction between genes and the environment. For example, *ZmGA3ox2* is a key candidate gene for a major quantitative trait locus influencing the plant height in maize [49], and brassinosteroids regulates cell elongation through modulation of gibberellin metabolism in rice [50]. Thus, we inferred that the heredity of pitaya flower length, perianth width, calyx tube width, the distance between stigma and anther, petal betalain content, petal and stigma number are mainly controlled by major genes and environmental conditions. The significant positive correlation between perianth width, calyx tube width, petal and stigma number may be from similar regulatory processes influencing these traits during flower growth and development. Understanding this interrelation is essential to grasping the genetic underpinnings and biological significance of these traits. 

### 4.3. The Genetic Effect of Stigmas

The stigma, serving as the primary interface for the initial interaction between the pistil and pollen grains, plays a pivotal role in pollination, influencing reproductive success and subsequent fruit production [51,52,53]. Moreira et al. found that more pollen deposition on the stigma resulted in higher rates of fruit setting and seed production [54]. Cross-pollination has been identified as a contributing factor to increase fruit size [55]. The height, expansion, and morphology (forked or unforked) of the stigma are directly correlated with pollen adhesion, thereby influencing pollination and fruit size. In this study, 73.5% of the F1 progenies of the ‘DH’ × ‘HHHQL’ cross combination had a lower stigma with unforked stigmas, similar to ‘DH’ pitaya traits. However, 65.1% of the F1 progenies of the ‘HHQL’ × ‘DH’ cross combination displayed a higher stigma with forked stigmas and, like the ‘HHQL’ pitaya trait, indicating a female inheritance pattern for the trait. The relative position of the stigma and anther significantly affects the success rate of pollination [56]. The stigma–anther relative position of the ‘DH’ pitaya is equal, leading to most of the F1 progenies in the ‘DH’ × ‘HHHQL’ cross combination exhibiting low stigmas. Therefore, selecting female parents with the desired stigma trait is advised for breeding forked stigmas in pitayas. In the F1 progenies of the ‘HHQL’ × ‘DH’ cross combination, the stigma–anther distance varied within the parental range, with some individuals surpassing the ‘HHQL’ pitaya; the higher-than-high parent rate was 47.1%. The F1 progenies of ‘DH’ × ‘HHHQL’ and ‘HHQL’ × ‘DH’ cross combinations had a high variation coefficient for this trait. Higher genetic heritability of stigma number was also detected in both F1 progenies, indicating a stronger genetic than environmental influence. 

A thorough comprehension of the genetic traits exhibited by both parents and their hybrid progenies in pitayas proves invaluable in obtaining offspring with the desired traits. This approach helps mitigate randomness in the breeding process, enhancing the efficiency of developing new varieties with the desired traits. This research has broadened the spectrum of phenotypic trait indicators for pitaya flowers, laying the groundwork for early selection and contributing to the exploration of high-quality new varieties. While flower traits are pivotal in understanding the pitaya’s phenotypic characteristics, delving into other major traits and factors is crucial for uncovering excellent genes and new germplasm resources in pitayas.

## 5. Conclusions

In this study, genetic variation, segregation patterns, and inheritance models were analyzed for flower-related traits of the F1 progenies from the ‘DH’ × ‘HHQL’ and ‘HHQL’ × ‘DH’ cross combinations. Pitaya flower traits showed a range of variations, and petal color is predominantly inherited from the ‘Dahong’ pitaya. The heritability of stigma numbers was higher compared to the calyx tube. The findings underscore the significant genetic diversity and major gene effects in pitaya flower traits, which are helpful for parental selection and targeted cross-breeding programs. The present study not only provides instructions for improving ornamental traits in further breeding programs, but also offers practical guidelines for the development of new pitaya varieties. 

## Figures and Tables

**Table 1:** Genetic analyses of flower traits of ‘DH’ × ‘HHQL’ and ‘HHQL’ × ‘DH’ cross combinations.

Flower Traits	Cross Combinations	Parents			F1 Progenies				
		HHQL	DH	MP	F ± *S*	*CV* (%)	*Ta (%)*	*Hm*	*R_Hm_* (%)
Flower length (mm)	D × H	229.54	302.97	266.26	254.34 ± 26.58	10.05	95.53	−11.29 **	−4.47
	H × D				269.09 ± 25.15	9.33	101.06	2.83 *	1.06
Perianth width (mm)	D × H	38.36	57.87	48.11	51.90 ± 6.01	11.57	107.88	3.79 **	7.88
	H × D				49.07 ± 4.92	10.02	101.99	0.96 **	1.10
Calyx tube width (mm)	D × H	18.72	29.67	24.19	24.33 ± 7.47	30.70	71.52	−9.68	−28.48
	H × D				21.26 ± 3.19	15.02	87.89	−9.85 **	−12.11
Distance between stigma and anther (mm)	D × H	22.21	0	11.1	2.88 ± 5.85	203.22	25.94	−8.22 **	−74.06
	H × D				32.19 ± 23.16	71.96	290.00	21.09 **	190.00
Betalain content (mg/100 g FW)	D × H	5.65	1.2	3.43	2.13 ± 1.80	84.60	62.10	−1.30 **	−37.90
	H × D				2.49 ± 1.64	66.13	72.59	−0.94**	−27.41
Petal number	D × H	12.0	22.0	17.0	16.72 ± 3.00	17.92	97.86	−0.28	−2.15
	H × D				13.47 ± 2.89	21.43	79.26	−3.35 **	−20.74
Stigma number	D × H	17.0	28.0	23.0	21.64 ± 2.51	11.62	96.18	−1.36 **	−3.83
	H × D				20.72 ± 2.97	14.35	90.08	−2.28 **	−9.92

* Indicates a significant difference at the 0.05 probability level; ** indicates an extremely significant difference at the 0.01 probability level. MP, mid-parent value; *CV*, coefficient of variation; *Ta*, genetic transmission ability of cross combination; *Hm*, mid-parent heterosis; *R_Hm_*, mid-parent heterosis rates.

**Table 2:** Akaike’s information criterion (AIC) under different genetic models in reciprocal progenies.

Model	Flower Length (mm)		Perianth Width (mm)		Calyx Tube Width (mm)		Distance between Stigma and Anther (mm)		Petal Betalain Content (mg/100 g FW)		Petal Number		Stigma Number	
	D × H	H × D	D × H	H × D	D × H	H × D	D × H	H × D	D × H	H × D	D × H	H × D	D × H	H × D
0MG	2344.12	3160.72	1603.52	2051.85	1711.89	1844.29	1590.67	3104.81	1002.97	1306.18	1259.75	1688.98	1170.77	1709.08
1MG-AD	2330.97	3155.03	1598.50	2037.49	1217.70	1844.40		3038.96	554.13	999.885	1258.30	1671.21	1166.52	1699.99
1MG-A	2329.94	3153.93	1596.84	2038.31	1223.22	1843.32	1091.66	3044.28	551.52	1052.40	1256.95	1669.26	1163.36	1700.90
1MG-EAD	2331.97	3160.29	1602.79	2045.65	1439.28	1845.95		3049.49	551.94	1007.09	1259.58	1678.85	1167.36	1703.53
1MG-NCD	2348.12	3163.74	1603.25	2052.14	1715.49	1846.36	1439.73	3085.58	903.10	1205.02	1261.61	1680.62	1174.77	1701.68
2MG-ADI	2351.10	3169.81	1610.48	2056.19	1193.38	1857.92	1343.41	3071.57	831.73	1104.45	1272.48	1683.09	1179.34	1713.17
2MG-AD	2324.89	3145.39	1600.10	2032.45	1185.18	1843.43	−12,002.29	−1904.88	−168.61	977.13		1672.80		
2MG-A	2328.97	3155.59	1597.05	2032.95	1188.64	1841.92	−19,589.97	3108.65	651.66	985.18	1257.56	1670.33		1702.89
2MG-EA	2327.04	3151.64	1597.27	2030.63	1186.92	1841.71	−14,152.0	−1398.03	648.13	1096.52	1256.36	1669.47	−1706.02	1694.80
2MG-CD	2347.41	3164.72	1607.53	2055.85	1715.88	1848.30	1594.67	3108.82	1006.97	1310.18	1263.76	1692.98	1170.81	1713.08
2MG-EAD	2345.41	3162.72	1605.53	2053.85	1713.88	1846.30	1592.67	3106.82	1004.97	1308.18	1261.76	1690.98	1169.07	1711.08

The AIC values of candidate genetic models are underlined.

**Table 3:** Tests for goodness-of-fit of selected model.

Characteristics	Cross Combinations	Model	U_1_^2^	U_2_^2^	U_3_^2^	nW^2^	Dn
Flower length (mm)	D × H	2MG-AD	0.0009 (0.9761)	0.0017 (0.9676)	0.0022 (0.9629)	0.0081 (1.0005)	0.0205 (>0.05)
	H × D	2MG-AD	0.0003 (0.9867)	0.0003 (0.9871)	0.0000 (0.9999)	0.008 (1.0006)	0.0144 (>0.05)
Perianth width (mm)	D × H	1MG-A	0.006 (0.9383)	0.0052 (0.9427)	0.0002 (0.9901)	0.025 (0.9896)	0.0289 (>0.05)
	H × D	2MG-EA	0.0013 (0.9708)	0.0006 (0.9799)	0.0017 (0.9672)	0.0268 (09855)	0.0225 (>0.05)
Calyx tube width (mm)	D × H	2MG-AD	0.0033 (0.9544)	0.0005 (0.9815)	0.0165 (0.8978)	0.0147 (0.9997)	0.0207 (>0.05)
	H × D	2MG-EA	0.0000 (0.9997)	0.0000 (0.9996)	0.0000 (0.9973)	0.0325 (0.9672)	0.0373 (>0.05)
Stigma relative to anther distance (mm)	D × H	2MG-A	72.5467 (0.0000)	54.2533 (0.0000)	12.4266 (0.0000)	12.7164 (0.0000)	0.5325 (<0.05)
	H × D	2MG-AD	0.1137 (0.7359)	0.0028 (0.9581)	2.2991 (0.1295)	0.1861 (0.2973)	0.112 (<0.05)
Betalain content (mg/100 g FW)	D × H	2MG-AD	0.6366 (0.4249)	0.3363 (0.5620)	0.5936 (0.4410)	0.1015 (0.5880)	0.0463 (<0.05)
	H × D	2MG-AD	0.0049 (0.9444)	0.0017 (0.9669)	0.1904 (0.6626)	0.0736 (0.738)	0.0418 (>0.05)
Petal number	D × H	2MG-EA	0.0014 (0.9699)	0.0001 (0.993)	0.0124 (0.9113)	0.2215 (0.2328)	0.0716 (>0.05)
	H × D	2MG-EA	0.0006 (0.9803)	0.0029 (0.9568)	0.0146 (0.9038)	0.3832 (0.0836)	0.0810 (<0.05)
Stigma number	D × H	2MG-EA	0.3124 (0.5762)	0.7375 (0.3905)	1.6138 (0.204)	0.6253 (0.0195)	0.1565 (<0.05)
	H × D	2MG-EA	0.0000 (0.9958)	0.0019 (0.9654)	0.0376 (0.8463)	0.3495 (0.1037)	0.0978 (<0.05)

The *p* values of each trait are shown in brackets.

**Table 4:** Estimation of genetic parameters for different traits with their optimal genetic model.

Genetic Parameters	Flower Length (mm)		Perianth Width (mm)		Calyx Tube Width (mm)		Distance between Stigma and Anther (mm)		Betalain Content (mg/100 g FW)		Petal Number		Stigma Number	
	D × H	H × D	D × H	H × D	D × H	H × D	D × H	H × D	D × H	H × D	D × H	H × D	D × H	H × D
M	261.207	271.055	51.942	49.116	25.310	24.170	3.176	28.483	2.627	3.042	16.79	13.48	21.51	20.72
*d*_a_	15.130	19.552	6.674	2.884	1.033	2.220	2.625	19.629	1.704	1.910	1.49	1.65	0.74	2.33
*d*_b_	8.918	−3.737			1.798		2.890	8.855	0.154	0.216				
*h*_a_	−10.876	4.488			−1.309			10.335	−1.537	−0.635				
*h*_b_	−3.179	−8.449			−1.071			3.038	−0.032	−0.023				
*I*														
*j*_ab_														
*j*_ba_														
*L*														
σ^2^_mg_	646.179	0.000	13.072	0.000	0.000	0.000	0.000	463.822	0.000	1.255	2.53	0.00	6.37	7.49
h^2^_mg_ (%)	91.108	0.000	36.080	0.000	0.000	0.000	0.000	86.452	0.000	46.404	27.77	0.00	100.00	84.67

**Table 5:** The main flower traits of the ‘DH’ and ‘HHQL’ pitayas.

Cultivars Traits	Petal Color	Stigma Color	Stigma Number	Stigma Split	Stigma–Anther Relative Position	Petal Number	Compatible/ Incompatible
DH	white	Yellow–green	28.0	absent	Equal	22.0	compatible
HHQL	red	faint yellow	17.0	present	Higher	12.0	incompatible

**Table 6:** SCoT primers used in this study.

Primer Names	Sequences (5′-3′)	Primer Names	Sequences (5′-3′)
SCoT-12	ACGACATGGCGACCAACG	SCoT-56	ACAATGGCTACCACTAGC
SCoT-13	ACGACATGGCGACCATCG	SCoT-58	ACAATGGCTACCACTAGG
SCoT-19	ACCATGGCTACCACCGGC	SCoT-61	CAACAATGGCTACCACCG
SCoT-21	ACGACATGGCGACCCACA	SCoT-62	ACCATGGCTACCACGGAG
SCoT-36	GCAACAATGGCTACCACC	SCoT-63	ACCATGGCTACCACGGGC
SCoT-42	ACCATGGCTACCACCGAT	SCoT-64	ACCATGGCTACCACGGTC
SCoT-47	ACAATGGCTACCACTGCC	SCoT-67	ACCATGGCTACCAGCGGC
SCoT-49	ACAATGGCTACCACTGCG	SCoT-73	CCATGGCTACCACCGGCT

## Data Availability

Data are contained within the article.
